# Leveraging AI to optimize vaccines supply chain and logistics in Africa: opportunities and challenges

**DOI:** 10.3389/fphar.2025.1531141

**Published:** 2025-02-10

**Authors:** Sulaiman Muhammad Musa, Usman Abubakar Haruna, Lukman Jibril Aliyu, Mubarak Zubairu, Don Eliseo Lucero-Prisno

**Affiliations:** ^1^ Department of Pharmaceutical Services, Ahmadu Bello University Teaching Hospital, Shika Zaria, Nigeria; ^2^ Faculty of Pharmaceutical Sciences, Ahmadu Bello University, Zaria, Nigeria; ^3^ Zipline, Kaduna, Nigeria; ^4^ UNICEF Country Office, Abuja, Nigeria; ^5^ Department of Global and Development, London School of Hygiene and Tropical Medicine, London, United Kingdom; ^6^ Office for Research, Innovation and Extension Services, Southern Leyte State University, Sogod, Southern Leyte, Philippines; ^7^ Center for University Research, University of Makati, Makati, Philippines

**Keywords:** Artificial intellingence, Africa, vaccines distribution, supply chain management, optimazation

## Abstract

Examining the current situation of the vaccine supply chain in Africa, the article highlights the importance of AI technologies while outlining the prospects and problems in vaccine supply chain management in Africa. Despite the significance of vaccinations, many African children are unable to receive them due to logistical challenges and a lack of infrastructure. AI has the potential to increase productivity by streamlining logistics and inventory management, but it is hampered by issues with data privacy and technology infrastructure. This perspectiveoffers ways for utilizing AI to enhance vaccine supply chains in Africa, citing successful experiences in Nigeria, Malawi, Rwanda, and Ghana as examples of AI’s advantages. In order to improve healthcare outcomes and immunization coverage in Africa, cooperation among stakeholders is stressed.

## 1 Introduction

The immunization program is designed to provide vaccines to children against vaccine-preventable diseases (VPD) such as polio and measles, targeting mostly children under 5 years of age. Vaccine-preventable diseases, however, remain a significant burden in Africa, with millions of children remaining unvaccinated. While vaccines play an important role in preventing infectious diseases and saving lives, they are fragile and sensitive to temperature changes. Maintaining their potency from production to the end user is crucial, but complex supply chain issues often lead to stockouts, waste, and inefficiency. Supply chain management of vaccines involves a systematic series of events, from procurement to availability at the last mile (Sarley et al., 2017).

The optimization of vaccine supply chains in Africa is a critical issue that has gained prominence, particularly in the wake of the COVID-19 pandemic. The continent faces unique challenges in vaccination efforts, including logistical hurdles, infrastructural deficits, and varying epidemiological landscapes. The World Health Organization has highlighted VPD remain a significant cause of morbidity and mortality among children in Africa, with disparities in vaccination coverage exacerbating these issues (Olutuase et al., 2022). The deployment of Artificial Intelligence (AI) technologies presents a promising avenue to enhance the efficiency and effectiveness of vaccine distribution systems across the continent (Subharun, 2023).

Artificial Intelligence (AI) refers to the ability of a digital computer or computer-controlled robot to perform tasks commonly associated with intelligent beings. The term is frequently applied to the project of developing systems endowed with the intellectual processes characteristic of humans, such as the ability to reason, discover meaning, generalize, or learn from experience.

AI can play a transformative role in vaccine supply chain management by improving forecasting, inventory management, and logistics. For instance, studies have shown that AI-driven analytics can optimize inventory levels and reduce stock-outs, thereby ensuring that vaccines are available when and where they are needed (Iwu et al., 2019). Furthermore, integrating AI into supply chain processes can facilitate real-time tracking and demand forecasting, which are essential for managing the complexities of vaccine distribution in diverse geographical contexts (Modgil et al., 2021). By leveraging predictive analytics and autonomous operations, AI technologies can enhance the resilience of vaccine supply chains, allowing for a more agile response to disruptions, such as those experienced during the COVID-19 pandemic.

Despite the potential benefits, implementing AI in vaccine supply chains in Africa is fraught with challenges. Issues such as data quality, infrastructure limitations, and the need for stakeholder engagement must be addressed to fully realize the advantages of AI technologies (Kazançoğlu et al., 2022; Modgil et al., 2021). Moreover, the complexity of vaccine supply chains, which includes cold chain requirements and the need for well-trained personnel, necessitates a comprehensive approach that considers both technological and human factors (Olutuase et al., 2022).

This paper, a perspective that aims to explore these opportunities and challenges in detail, provides a framework for understanding how AI can be effectively harnessed to optimize vaccine supply chains in Africa. And also examines the current state of vaccine distribution, identifying best practices from countries that have successfully integrated AI technologies, and proposing actionable strategies to enhance supply chain resilience and efficiency.

### 1.1 Current state of vaccine distribution in Africa

The distribution of vaccines in Africa has been a multifaceted challenge, exacerbated by the COVID-19 pandemic. Despite the global urgency for vaccination, Africa has faced significant hurdles in both the acquisition and distribution of vaccines. Reports indicate that as of early 2021, many African countries had administered fewer than 10 doses of COVID-19 vaccines per 100 people, starkly contrasting with higher rates in regions like the United States and Europe, where vaccination campaigns began much earlier (Sen-Crowe et al., 2021). This disparity highlights systemic issues in the supply chain and logistics that have historically plagued vaccine distribution across the continent. Many countries procure vaccines through centralized processes, such as UNICEF’s initiatives, which ensure quality vaccines at negotiated prices. Organizations like GAVI also provide financial support to negotiate lower costs for low- and middle-income countries (Duijzer et al., 2018).

Vaccines must be transported under strict cold chain conditions (+2 to +8°C), presenting significant logistical challenges due to inadequate infrastructure and electricity shortages in many regions (World Health Organization, 2018). Solar-powered equipment has been introduced to mitigate these challenges (Aina et al., 2017). For instance, Nigeria operates a centralized distribution system where vaccines are sent from national stores to zonal cold stores quarterly, and further distributed to local governments monthly.

Generally, the logistical Challenges Currently Faced in Vaccine Rollout in Africa includes:

1. Dedicated fund for last-mile vaccine delivery.
2. Lack of coordination among vaccine supply chain stakeholders.
3. Lack of cold chain infrastructure.
4. Frequent active cold chain equipment breakdown.
5. Difficulties in transporting vaccines to hard-to-reach areas.
6. Lack of last-mile vaccine visibility tool.
7. Inadequate human resource.

## 2 Case studies of successful vaccine rollout in Africa

### 2.1 Nigeria

In Nigeria, several states have successfully implemented last-mile vaccine delivery through partnerships involving the Bill and Melinda Gates Foundation (Gates Foundation, 2022), the Aliko Dangote Foundation, the Gavi HSS Fund (Gavi, The Vaccine Alliance, 2024), and UNICEF according to the record at National Primary Healthcare Development Agency (NPHCDA, 2024). The following Table 1 summarizes some states and their funding sources for last-mile delivery:

**TABLE 1:** Example of Nigerian states and thier source of funding vaccine rollout.

S/N	State	Source of funding
1	Bauchi	Tripartite MOU
2	Bayelsa	GAVI HSS
3	Borno	Tripartite MOU
4	Gombe	GAVI HSS
5	Jigawa	GAVI HSS
6	Kaduna	Tripartite MOU
7	Kano	Tripartite MOU
8	Katsina	GAVI HSS/UNICEF
9	Kebbi	GAVI HSS
10	Lagos	State Government
11	Niger	GAVI HSS
12	Oyo	State Government
13	Sokoto	Tripartite MOU
14	Taraba	GAVI HSS
15	Yobe	Tripartite MOU
16	Zamfara	GAVI HSS/UNICEF

Source: National Primary Healthcare Development Agency (NPHCDA), Bill and Mellinda Gate Foundation (BMGF), GAVI, the global vaccine alliance; UNICEF, abuja office.

Successes:

1. Increased vaccine availability at service points.
2. Improved stock visibility for better management.
3. Reduced out-of-pocket expenses for health workers.
4. Shorter patient wait times at service points.
5. Greater ownership and confidence in vaccine management by health facilities.

Limitations:

1. Deliveries often stop at equipped health facilities or LGA cold stores.
2. Insufficient funds for equipping health facilities.
3. Delays in fund disbursement impacting timely deliveries.
4. Instances of overstocking leading to potential expiration.

### 2.2 Malawi

Malawi employs a mixed distribution system for vaccines, utilizing both pull and push methodologies. The regional stores pull vaccines from the national store quarterly, while district stores pull monthly to mitigate spoilage due to electricity shortages (Sustainable Energy for All Malawi Integrated Energy Plan: An Overview, 2022).

### 2.3 Distribution mechanism

Pull System: Regional stores pull from national stores quarterly; district stores pull from regional stores monthly.

Push System: Vaccines are pushed from district levels to health facilities monthly, with outreach staff delivering vaccines to remote locations.

## 3 Opportunities in integrating AI technology in vaccine supply chain optimisation

The integration of Artificial Intelligence (AI) presents significant opportunities to optimize vaccine supply chains by addressing existing challenges:

### 3.1 Supply chain optimization

Artificial Intelligence can transform and improve the efficiency of the vaccine supply chain by reducing operations and ensuring on-time and in-full delivery of vaccines to service delivery points (Munasinghe and Halgamuge, 2023). The current vaccine supply chain system is linear and often reactive, leading to increased stock-out rates and expired vaccines because of oversupply and wastage of vaccines. AI can help streamline the communication channel among the supply chain stakeholders.

### 3.2 Predictive analytics/forecasting methods

Accurate vaccine needs estimation is important for effective vaccine logistics and supply chain management (Sarley et al., 2017). However, the complexity in predicting absolute vaccine needs occurred because of fluctuations in population growth, disease outbreaks and many vaccination campaigns to close the immunization coverage gap. Accurate estimation of all stock requirements is necessary to avoid shortage and prevent wastage from excessive orders.

Vaccines need can be estimated using three methods:

1. Target population-based estimation.
2. Previous consumption-based estimation.
3. Previous vaccination number-based estimation.

AI technology can synthesize and analyze historical immunization data and identify clear trends to predict future vaccine demand accurately. This will help immunization supply chain personnel to use data to plan the allocated vaccines by taking into consideration the fluctuation in vaccine demand by different regions.

### 3.3 Innovative delivery mechanism

AI technology can optimize the delivery route, reducing transportation time and fuel consumption by identifying the most efficient delivery path for vaccine delivery (Sato et al., 2023). AI can help in shifting the distribution of vaccines from the current push method with the use of previously forecasted data to a real-time informed push-plus allocation based on the real-time demand of the health facility, the use of AI will help in having real-time visibility into the consumption data in the health facility and optimize the vaccine allocations (Munasinghe and Halgamuge, 2023).

### 3.4 Inventory management and monitoring and evaluation optimization

Inventory management, stock monitoring and evaluations is one of the critical components of vaccine management. Proper management of stocks prevents stock out, overstocking and wastage of vaccines.

Artificial intelligence can automate the tracking of vaccine levels in real-time from all levels of the supply chain and update and automate the recording of vaccine stock at the last mile. This automation will help supply chain managers to update the real-time changes in vaccine demand. Vaccine wastage can be reduced using AI-driven predictive analysis by identifying the near-expiry vaccines and those that are likely to expire. AI-driven analytics can be used to monitor and evaluate key supply chain performance indicators and make informed data-driven decisions (Munasinghe and Halgamuge, 2023).

## 4 Challenges and barriers to AI integration in vaccine supply chain optimization

The integration of AI into vaccine supply chain management encounters several substantial challenges that can hinder effective implementation. These challenges can be categorized into distinct areas:

### 4.1 Infrastructure limitations

Technological infrastructure: Many healthcare systems, particularly in resource-limited settings, lack the essential technological infrastructure necessary for AI integration. This includes insufficient hardware, software, and network capabilities critical for effective AI operations (Olutuase et al., 2022; Nair et al., 2024).

Data quality and availability: AI requires access to high-quality, comprehensive datasets to function optimally. However, in numerous regions, data collection systems are often fragmented or absent, resulting in significant gaps that hinder the effectiveness of AI applications (Seelos et al., 2024; Džubák and Hanzelka, 2022).

### 4.2 Data privacy and security

Privacy concerns: The need for AI systems to access sensitive health information raises significant privacy issues. Ensuring the security of patient data is a major challenge as these systems can be vulnerable to data breaches and unauthorized access (Shen et al., 2024; Mooghali et al., 2024).

Regulatory compliance: Compliance with stringent data protection regulations such as GDPR in Europe and HIPAA in the United States complicates AI implementation. These regulations demand rigorous protocols for data handling and storage, which can slow down the integration process (Kudrenko, 2024; Ahmed et al., 2023).

### 4.3 Technical expertise

Lack of expertise: There is a notable shortage of professionals skilled in developing, implementing, and maintaining AI systems within healthcare environments. This gap includes both technical experts in AI and healthcare professionals trained to effectively utilize these technologies (Kudrenko, 2024; Bates, 2024).

Training and education: Ongoing education and training programs are vital for equipping healthcare workers with the necessary skills to leverage AI effectively. However, such programs are often insufficient or lacking entirely (Seelos et al., 2024; Bates, 2024).

### 4.4 Stakeholder trust and collaboration

Trust in AI systems: Establishing trust among healthcare providers, patients, and other stakeholders is essential for successful AI integration. Concerns about the reliability and transparency of AI systems can hinder their acceptance and widespread adoption (Hu et al., 2022; Li et al., 2023).

Collaboration barriers: Successful AI implementation requires collaboration among various stakeholders, including healthcare providers, IT professionals, and policymakers. Misalignment of goals and poor communication can significantly impede progress (Seelos et al., 2024).

### 4.5 Deficiencies in governmental support

Policy and regulation: The lack of clear policies and regulatory frameworks governing the use of AI in healthcare creates uncertainty that can slow down implementation efforts. Governments must establish guidelines that promote innovation while ensuring safety and efficacy (Seelos et al., 2024; Ahmed et al., 2023).

Funding and investment: Adequate funding is crucial for supporting AI initiatives. Many healthcare systems, particularly in low-income countries, face financial constraints that limit their ability to invest in necessary AI technologies (Kudrenko, 2024; Nair et al., 2024).

## 5 Case studies of successful AI implementation in vaccine supply chain management

Despite these challenges, several countries have successfully integrated AI into their vaccine supply chain management:

### 5.1 Case study 1: Viebeg technologies in Rwanda

Viebeg Technologies operates in Rwanda, utilizing AI to manage supply chain processes such as shipping, warehousing, distribution, and inventory management. This approach ensures that healthcare facilities maintain precise stock levels of medical supplies, thereby reducing stock-outs and shortages (African Development Bank Group, 2024).

### 5.2 Case study 2: Zipline’s drone delivery service

Zipline employs autonomous drones to deliver vaccines and medical supplies across Ghana, Rwanda, Nigeria, Kenya, and Côte d’Ivoire. By integrating AI into its operations as a digital-first company, Zipline has significantly reduced delivery times while improving vaccine availability. The drones use advanced algorithms to optimize delivery routes, ensuring efficient distribution even to remote locations (Seelos et al., 2024; Addy, 2023).

### 5.3 Case study 3: electronic vaccine intelligence network (eVIN) in India

India’s eVIN employs AI algorithms to monitor real-time data on vaccine stocks, storage temperatures, and distribution logistics across its vast healthcare landscape. This system has streamlined vaccine supply chain management, achieving an impressive 80% reduction in stockouts (UNDP n.d.).

## 6 Future directions

The current study highlights the need for continuous improvement in vaccine distribution systems across Africa to enhance healthcare outcomes for vulnerable populations through:

▪ Infrastructural development in the technological sector.
▪ Enhancing data quality, availability and security.
▪ Investing technical expertise.
▪ Building trust, support and collaboration among stakeholders, including governments at all levels.
▪ Implementing of AI Technologies in vaccine rollout.

**FIGURE 1:**
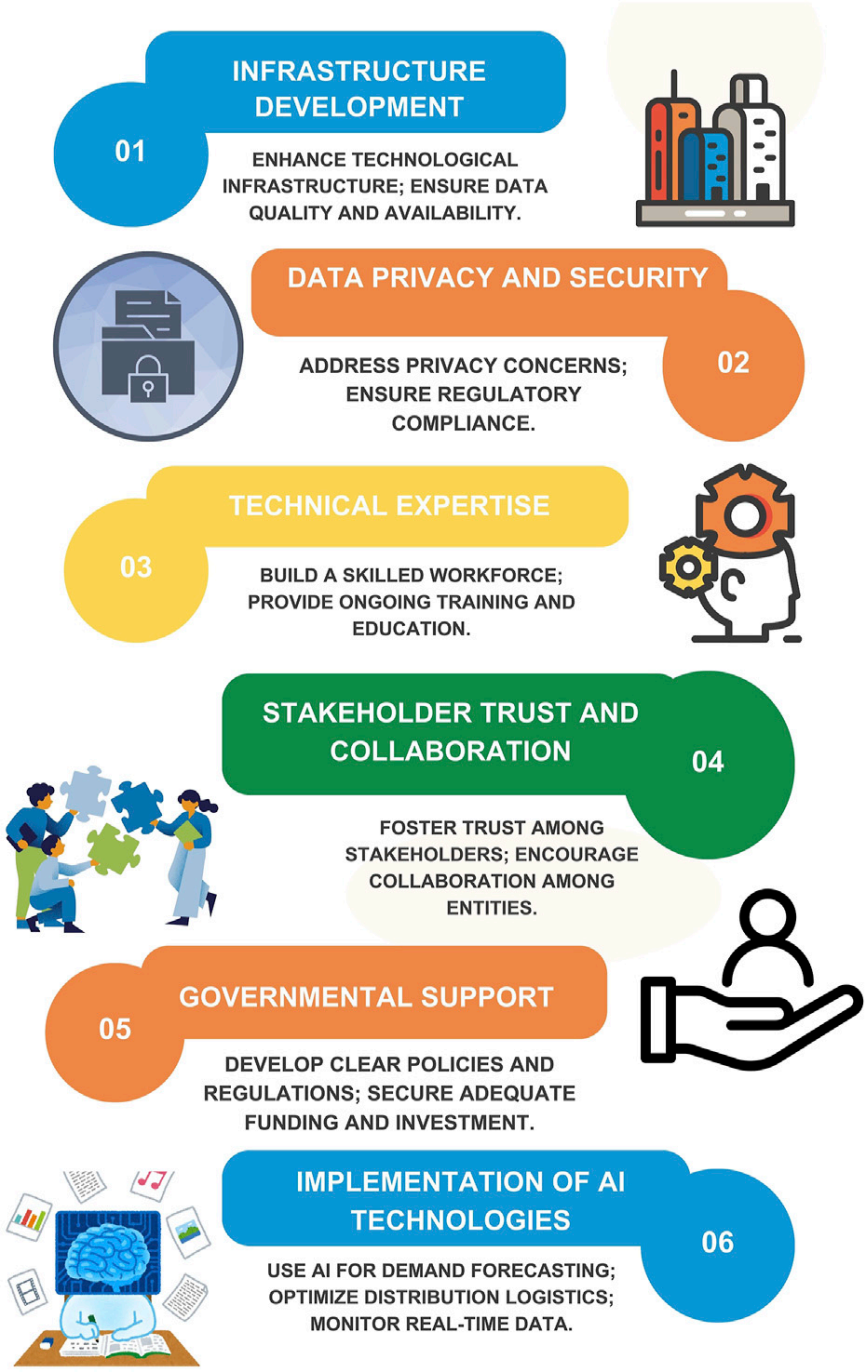
Framework for successful integration of AI in vaccine rollout.

Figure 1 above indicated a framework that can be utilised to achieve successful implementation of AI in Vaccine roll out in Africa.

## 7 Conclusion

Integrating AI technology into vaccine supply chain optimization presents significant challenges including infrastructure limitations, data privacy concerns, technical expertise deficits, stakeholder trust issues, and governmental deficiencies. However, successful case studies from countries like Rwanda and India illustrate that addressing these barriers through strategic investments and collaboration can enhance efficiency in vaccine distribution, inventory management, and real-time monitoring. By embracing AI technologies across Africa, countries can significantly improve their vaccine supply chain management processes—ultimately leading to better healthcare outcomes for their populations.

## Data Availability

The original contributions presented in the study are included in the article/supplementary material, further inquiries can be directed to the corresponding author.
